# The cost of clinical management of SARS-COV-2 (COVID-19) infection by level of disease severity in Ghana: a protocol-based cost of illness analysis

**DOI:** 10.1186/s12913-021-07101-z

**Published:** 2021-10-18

**Authors:** Hamza Ismaila, James Avoka Asamani, Virgil Kuassi Lokossou, Ebenezer Oduro-Mensah, Juliet Nabyonga-Orem, Samuel Kaba Akoriyea

**Affiliations:** 1 Ghana Health Service, Headquarters Office, Private Mail Bag, Ministries, Accra, Ghana; 2 World Health Organisation, Regional Office for Africa, UHC Life Course Cluster, Intercountry Support Team for Eastern and Southern Africa, Zimbabwe, South Africa; 3 ECOWAS Regional Centre for Disease Surveillance and Control, Abuja, Nigeria; 4 Ghana Health Service, Ga East Municipal Hospital, Accra, Ghana

**Keywords:** Cost of COVID-19, Cost of illness analysis, COVID-19, Case management of COVID-19, Bottom-up, Point of care resource use, Costing

## Abstract

**Background:**

As the global strategies to fight the SARS-COV-2 infection (COVID-19) evolved, response strategies impacted the magnitude and distribution of health-related expenditures. Although the economic consequence of the COVID-19 pandemic has been dire, and its true scale is yet to be ascertained, one key component of the response is the management of infected persons which its cost has not been adequately examined, especially in Africa.

**Methods:**

To fill gaps in context-specific cost of treating COVID-19 patients, we adopted a health system’s perspective and a bottom-up, point of care resource use data collection approach to estimate the cost of clinical management of COVID-19 infection in Ghana. The analysis was based on the national protocol for management of COVID-19 patients at the time, whether in public or private settings. No patients were enrolled into the study as it was entirely a protocol-based cost of illness analysis.

**Result:**

We found that resource use and average cost of treatment per COVID-19 case varied significantly by disease severity level and treatment setting. The average cost of treating COVID-19 patient in Ghana was estimated to be US$11,925 (GH¢68,929) from the perspective of the health system; ranging from US$282 (GH¢1629) for patients with mild/asymptomatic disease condition managed at home to about US$23,382 (GH¢135,149) for critically ill patients requiring sophisticated and specialised care in hospitals. The cost of treatment increased by some 20 folds once a patient moved from home management to the treatment centre. Overheard costs accounted for 63–71% of institutionalised care compared to only 6% for home-based care. The main cost drivers in overhead category in the institutionalised care were personal protective equipment (PPEs) and transportation, whilst investigations (COVID-19 testing) and staff time for follow-up were the main cost drivers for home-based care.

**Conclusion:**

Cost savings could be made by early detection and effective treatment of COVID-19 cases, preferably at home, before any chance of deterioration to the next worst form of the disease state, thereby freeing up more resources for other aspects of the fight against the pandemic. Policy makers in Ghana should thus make it a top priority to intensify the early detection and case management of COVID-19 infections.

**Supplementary Information:**

The online version contains supplementary material available at 10.1186/s12913-021-07101-z.

## Background

In December 2019, an unknown respiratory illness was reported in the Hubei Province of China, a disease later named COVID-19 and caused by Severe Acute Respiratory Syndrome Coronavirus type-2 (SARS-COV-2) [1]. The disease subsequently spread globally and was declared a pandemic by the World Health Organization (WHO) in March 2020 [2]. As of 17 September 2020, 29,679,284 cases were confirmed globally with 936,521 deaths while the WHO African Region recorded 1,127,164 confirmed cases and 24,294 deaths [3, 4].

The global response measures at the time was not uniform across countries but notably centred on centred on travel restrictions, evolving into partial and complete lockdowns to drastically limit physical interactions with the intent of curbing the spread of the virus alongside aggressive testing and treatment of infected persons [5]. Other public health and social measures included encouraging individuals to observe physical distancing of 1–2 m, use of face masks (or face coverings) when outside of home, regular hand washing or disinfection and isolation when one was exposed to someone infected. The World Health Organisation issued varied guidelines (updated from time to time) and countries adapted it to their local context.

In the initial phase, the Ghanaian authorities, focused on preparedness and response planning in which the relevant WHO Guidelines were adapted into a country strategy [6]. The strategy prioritised setting up of isolation/treatment and/or quarantine centres, procurement and distribution of personal protective equipment (PPEs), discouraging non-essential travel into Ghana, temporarily suspending all foreign travels for public officials and mobilizing $US100 million towards implementation of the country’s preparedness and response strategy [7–10]. When Ghana recorded its first case of COVID-19 on 12th March 2020, it initiated an aggressive contact tracing program leading to the detection of 132 cases before the end of March 2020 [10, 11] and community spread had been established across all 16 administrative regions before end of June 2020, but majority of cases remained in the two most urbanised regions (Greater Accra and Ashanti). The case count increased dramatically to 45,655 by mid-September 2020 although mortality rate remained markedly lower than 1% [12]. In addition to making the wearing face mask compulsory, the country closed its borders, closed down schools and adopted what was termed as the “test, treat and trace (3-Ts) approach”. The 3-Ts approach approach involved rapid and enhanced contact tracing, general surveillance and testing measures as well as case management in designated health facilities [13, 14]. Laboratory testing sites were increased from one to 10; medical drones deployed for sample quick transportation; COVID-19 Tracker mobile application launched;  additional 45,107 health workers (1000 as temporal contact tracers) employed;50% salary incentive plus tax exemptions provided to its health workforce; and over 7000 health facilities designated as treatment centres [15, 16].

Although the economic consequence of the COVID-19 pandemic had been dire, and its true scale yet to be ascertained, one key component of the response was the management of infected persons which its cost has not been adequately examined. Earlier studies in China estimated the mean cost of treating COVID-19 patient from a health system perspective to be about US$6827 which roughly amounted to US$ 0.49 billion for the entire clinical management of COVID-19 in China [17]. Using a similar health system perspective, the World bank generated regional averages of US$3.65 for a mild case and US$15.56 for a hospitalized patient in sub-Saharan Africa [18]. However, these estimates were premised on effective prevention measures (wide scale and intensive social distancing) which were not met.

There are very limited country-specific analyses that estimate the cost of treating COVID-19 cases from health systems perspective in Africa. One Kenyan analysis estimated the cost of treating COVID-19 patients to range from US$282 in home management to US$5707 in critical care settings [19]. In another attempt to estimate the probable cost of the entire response to COVID-19, Rueda et al. extrapolated data from costing of other diseases in South Africa, Ethiopia, and Pakistan to low-and-middle-income countries. The authors estimated the cost of treatment to be between US$147 in home-based management and US$1082 per day for critically ill patients managed in resource-intensive settings [20]. However, as the characteristics of the pandemic evolved, so did the global strategies, which ultimately impacted on the case management and overall response of countries. The wide variations in case management protocols between countries have made it even imperative for context-specific estimation of the cost of treatment as part of the needed evidence towards the adoption of sustainable policies and priorities on COVID-19 interventions. The context-specific evidence gap in the cost of treating COVID-19 patients tend to slow down rapid resource planning, hence the analysis sought to fill the gap in the context of Ghana but could be replicated in other settings.

We adopt a health system’s perspective and a bottom-up, point of care resource use data collection approach to estimate the protocol-based cost of clinical management of COVID-19 infection in Ghana.

## Methods

**Fig. 1:**
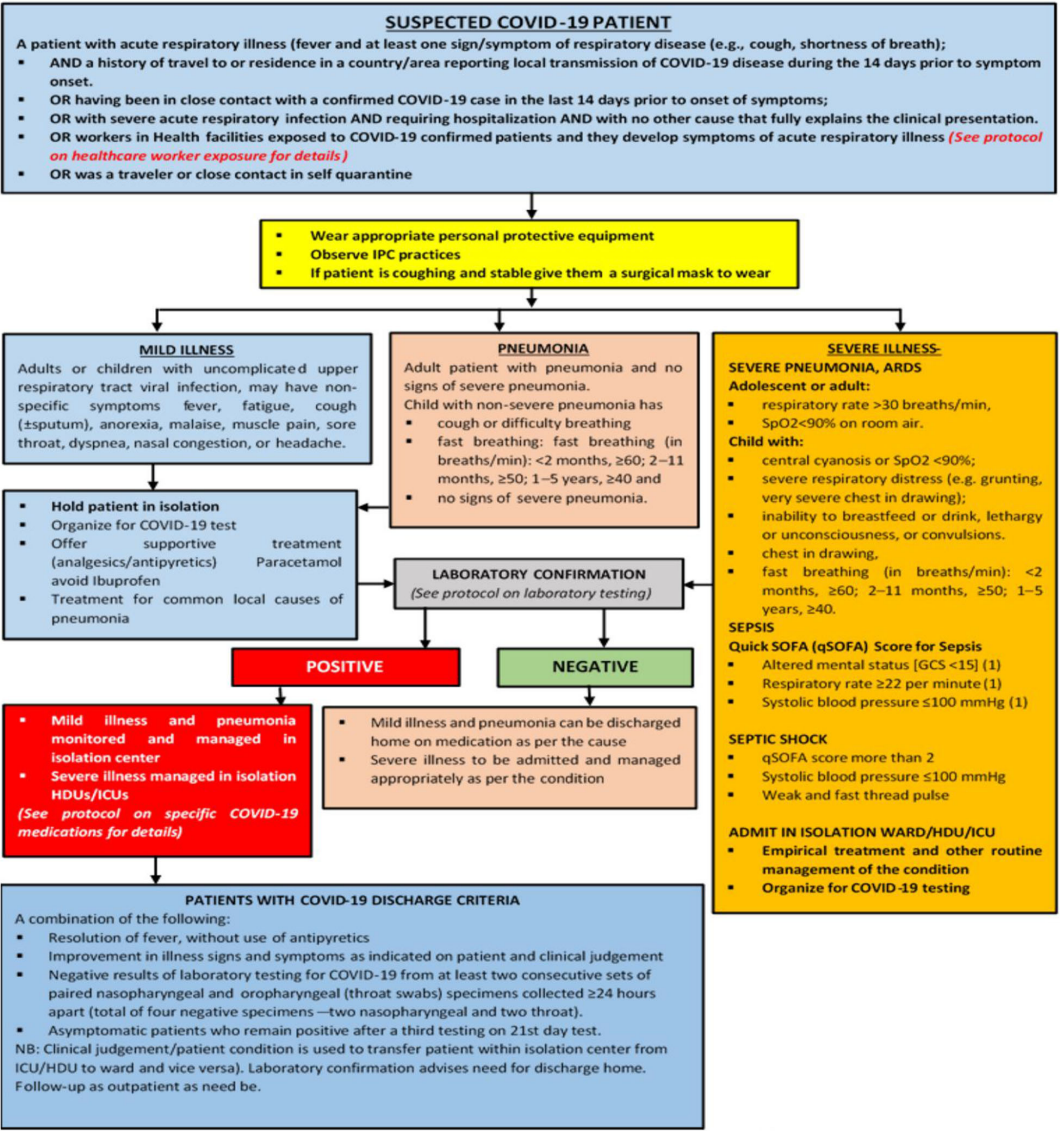
Clinical management protocol in use as of September 2020. Source: Ministry of Health. COVID-19 management guidelines

**Fig. 2:**
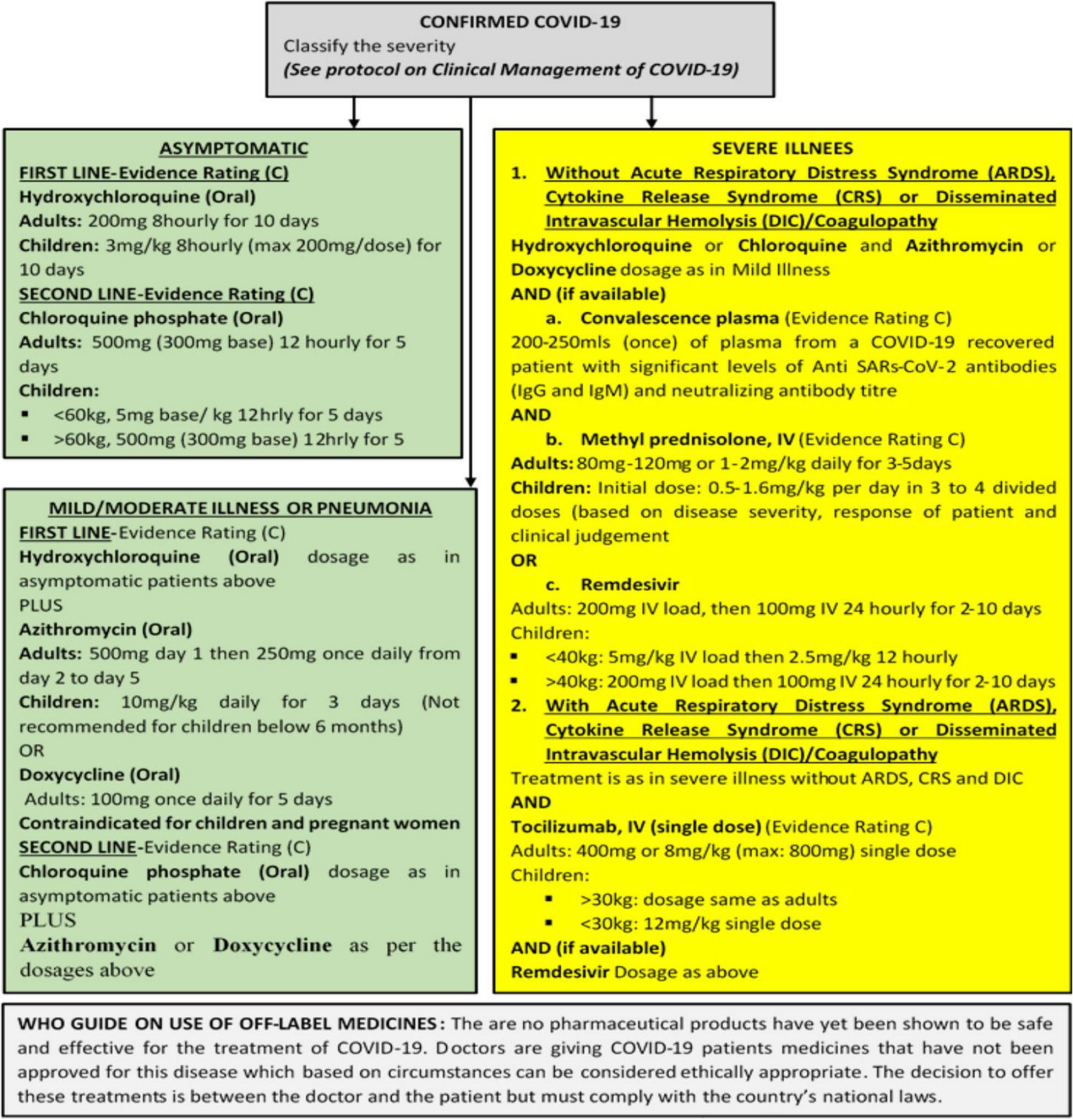
Specific guide on medicines to be used. Source: Ministry of Health. COVID-19 management guidelines

We employed a costing approach guided by the costing framework proposed by Drummond [21] and based on the approved national COVID-19 treatment protocol for Ghana (see summary in Figs. 1 and 2) [6]. We adopted a bottom-up, point of care resource use data collection approach. Resource use for each type of patient (according to disease severity) was identified and quantified using the case management protocol in use as of September 2020 and with the advice of clinical experts in the frontlines of protocols development and COVID-19 treatment in the country. No patients were enrolled into the study and hence, no patient characteristics were analysed or described. However, protocol-based cost analyses have a good place in planning and resource mobilisation, as well as guiding updates and implementation of the protocol as they make available resource use implications to clinicians and policy makers. It is in this light that this paper sought to adopt a protocol-based analysis when enrolling COVID-19 patients was ethically not permissible at the time. The protocol-based cost analysis was conducted between August and September 2020.

### The perspective of cost

The Ministry of Health fully bore the cost of COVID-19 treatment; hence the Ministry’s perspective of the cost was adopted for this analysis. Costs related to loss of income to patients were not considered, as were those  losts of revenue to health facilities due to reduced utilization of other routine services arising from the suspension of such services or patients not demanding for them for fear of the COVID-19 pandemic.

### Resource use identification and quantification per patient by the level of severity

The COVID-19 treatment protocol of Ghana as of September 2020 was reviewed together with clinicians to identify resource use elements for the different levels of disease severity and setting of treatment (home or institutionalised care) which culminated in the development of resource use identification template. The resource template was used to collect data, with the assistance of frontline clinicians, on the resources used to treat each type of patient (by the level of disease severity and treatment setting), and the quantity of the resources used. The identified resource use with quantities was validated by two of the country’s leading experts in COVID-19 case management and further reviewed by the case management Team Lead of the West African Health Organisation (WAHO). The resource use was categorised into five [5]: overheads (patient accommodation, utilities, feeding and toiletries, as well as set of personal protective equipment used by the health professionals), investigations, medications, in-patient care and human resources (staff time). In the case of home isolated patients, we considered costs relating to provided items (thermometer, cost of visits and staff time). Ghana’s treatment protocol for COVID-19 specified that all moderate, severe or critically ill patients be treated in hospitals while those with no or mild symptoms are supported to manage at home or at isolation centres where the home environment is deemed inconducive for isolation.

#### Resource use and cost drivers by the level of severity

Patients that received institutionalised care at isolation centres or hospitals were transported from their homes or point of referral to the treatment centre or hospital and sent back upon discharge. This was an essential part of the overhead cost alongside patient accommodation, feeding and toiletries, as well as set of personal protective equipment used by the health professionals. The resource need differentials in the overhead category were driven by the average length of stay, which was for up to 21 days for severely or critically ill patients and 19 days for the rest. Home managed patients received a thermometer for self-monitoring and were paid visits by clinicians (staff time) which formed the overhead costs from the health system perspective.

Resources needed for investigations and monitoring of patient prognosis included materials for sample taking and reagents like test kits for SARS-COV-2 test, full blood count, blood gases, chemistries, and coagulation profile. Other investigative procedures included x-rays, computer tomography (CT) scans, electrocardiogram (ECG) and ultrasound scans for pregnant women. The resources needed for, and frequency of, these investigations varied markedly depending on the severity of the disease. For instance, while all these were necessary for critically ill patients, analysing blood gases were not indicated for those classified as severe unless they were put on a ventilator (where oxygen saturation was less than 90% with a continuous downward trend despite optimal oxygenation or when significant lung changes were detected on x-ray or CT scan). Similarly, blood gases, CT-Scan, chemistries, ECG and coagulation profile were not included in the monitoring protocol for those with mild illness or those that were asymptomatic. For patients managed at home, only routine temperature checking, and the SARS-COV-2 test were needed.

Another category of resource use was medications, which the treatment protocol outlined the use of vitamin C with zinc, hydroxychloroquine (or chloroquine) and azithromycin for all patients regardless of the treatment settings. Patients with severe illness or those who required high dependency or intensive care received additional antibiotics such as ceftriaxone and thrombolytics (commonly enoxaparin).

Resources needed to facilitate the in-patient care of all institutionally managed patients included oxygen for patients who experienced difficulty in breathing, and mechanical ventilation for critically ill patients, patients with severe symptoms and oxygen saturation of 90% or less, as well as for in-patients with significant lung changes on x-ray or CT scan. The use of oxygen and mechanical ventilation was concomitantly associated with the use of syringes, needles, oxygen masks, endotracheal tubes, among others.

The health workforce needed for the management of each case depended on the level of severity and availability of other resources. For example, severely ill patients required up to 4 h of medical specialists’ time and 6 h of nurses’ time per day – of various skill-mix. Additional file 1: **Supplementary appendix 1** provides details of the resource needs identified for each level of the disease severity and the associated unit cost. The rest of the analysis was based on these resource use and unit costs.

### Assigning unit cost for each unit of resources used

The unit cost of the resources used in treating COVID-19 patients was triangulated from Ghana’s National Health Insurance Scheme (NHIS) price list for medicines and services [22, 23], average prices from the Public Procurement Authority [24], the Government salary structure for public sector health workers [25] and invoices of procured goods that were that hitherto were not regularly procured (example Personal Protective Equipment, PPEs). Where the unit costs could not be obtained from these sources, local open market prices were used except for resources that were not on sale in the local market where the international prices were adopted.

### Cost estimation per patient tenement

Using the resources identified and quantified for each patient type, and the unit cost, the cost of treatment per patient (by the level of severity) was computed using the following formula:

$$ Total\ cost\ of\ treatment\ for\ patient\ i={\sum}_j\left({Resource}_{ij}x\ {Unit\ cost}_j\right) $$

Where:

- Resource_ij_ is the amount of resource j used by patients with disease severity i
- Unit cost_j_ is the unit cost for resource j

We compared the cost of treatment by the different categories of resource use within the same level of disease severity and across the different levels of disease severity as well as treatments settings – home-based care or institutionalised care. The estimated costs were not discounted or adjusted for inflation since they were cross-sectional with no long-term extrapolation. The official exchange rate of 5.78 Ghana Cedis to US$1 (as of end of August) was used for converting the cost from the local currency (Ghana Cedis) to the United States Dollar.

## Result

### Average cost of treatment per COVID-19 case – by level of disease severity

The cost of treating COVID-19 patients in Ghana irrespective of the setting and level of severity from the perspective of the health system ranged from US$282 (GH ¢1629) to US$23,382 (GH ¢135,149), with an average of US$11,925 (GH ¢68,929). For patients that were treated in hospitals or treatment centres, the overhead cost, notably for PPEs and Transportation, were the main cost drivers, followed by the cost of in-patient care (see Table 1 for cost summary in USD and supplementary Tables S1 – S5 for resource use and unit cost in local currency unit, the Ghana Cedis). For home management, the main cost drivers were investigations (COVID-19 testing) and staff time. As shown in Table 2, overhead cost on average accounted for 55% (6–71%) of the cost of clinical management COVID-19 cases in Ghana, followed by in-patient care which accounted for 19% (17–22%). Across all cases, the third cost driver was staff time, accounting for 18% (4–42%) of the treatment cost, followed by investigations which accounted for 11% (1–47%) of the total cost. Interestingly, medications constituted only 2% (0.02–5%).

**Table 1:** Estimated cost of COVID-19 treatment by the level of severity and treatment setting (in United States Dollars, USD)

Cost Category	Cost of home management (USD)	Estimated cost by level of disease severity for institutionalised care (USD)				Average
		Mild	Moderate	Severe	Critical	
In-patient care	–	1259	1269	3546	4587	4066
Investigations	132	147	340	277	489	277
Medications	14	14	89	199	335	130
Overheads	17	4072	6701	13,276	14,660	7745
Staff time	118	215	1552	3007	3312	1641
Total	282	5707	9952	20,305	23,382	11,925

Note: the analysis presented in the table was based on the COVID-19 treatment protocol. No patient data is reported in this table

**Table 2:** Proportional cost distribution by cost category, level of severity and treatment setting

Cost Category	Home management	Level of Disease Severity for Institutionalised Care			
		Mild	Moderate	Severe	Critical
In-patient care	0%	22%	13%	17%	20%
Investigations	47%	3%	3%	1%	2%
Medications	5%	0.2%	1%	1%	1%
Overheads	6%	71%	67%	65%	63%
Staff time	42%	4%	16%	15%	14%
Total	100%	100%	100%	100%	100%
Cost difference (US$)		5425	4245	10,353	3078
% Increase disease severity to another		1925.2%	74.4%	104.0%	15.2%

Note: the analysis presented in the table was based on the COVID-19 treatment protocol. No patient data is reported in this table

It is worth noting that there was a wide range in the proportion of cost attributable to overheads, staff time and investigations. For example, there was a 65% cost differential between the overhead cost of patients managed at home and those managed in institutional settings; and across the different levels of disease severity of those that were institutionally managed. Eighty one percent (81%) of the overhead costs for institutionally-managed patients were attributable to PPEs, 28% for accommodation and utilities, and 3% for transportation. To put this in perspective, critically ill patients who spent about 21 days in hospitals consumed up to 210 sets of PPEs for the duration of their stay as compared to 38 sets of PPEs consumed by patients with mild symptoms with an average length of stay of 19 days. Similarly, several investigations (and repeat investigations) were required for patients with increasing levels of severity, thereby accounting for 46% in the cost variation observed for investigations. Also, as level of severity increased, the expertise and number of health workforce (staff time) needed to address the patient health problem increased, hence a US$10,588 difference between the staffing costs of treating mild and critically ill patients. This represents a 186% difference in the staffing costs between the extremes of the disease when managed in institutional settings.

### Cost differences in various levels of disease severity and treatment settings

Ghana’s case management protocol for COVID-19 allowed for patients without obvious symptoms and those with mild symptoms to be managed at home, if in the assessment of the clinician, the conditions necessary for effective management at home were met. These cases required fewer resources classified in the overhead’s category, fewer clinical investigations, health workforce expertise and medications.

Once a patient moved from home management to the treatment centre, the cost of treatment increased by at least 20 folds. The criteria for managing a COVID-19 patient in a treatment centre/hospital included mild and asymptomatic cases whose home environment were evaluated to be unconducive for effective treatment. These patients were usually kept in COVID-19 isolation/treatment centres or specially designated areas in hospitals. Additionally, all moderate, severe and critically ill patients were managed in hospitals with the appropriate capacities to address their health needs. The cost of managing a patient with mild symptoms in isolation/treatment centre was estimated to be US$5707 (GH ¢32,985), with overheads accounting for about 71% of this cost whiles in-patient care and staff time accounted for 22% and 4% respectively.

The cost of treating patients with severe symptoms and the critically ill was about US$20,305 (GH ¢117,361) and US$23,382 (GH ¢135,149), respectively. Thus, deteriorating from moderate to severe resulted in about US$10,353 (104%) additional cost, and a relatively marginal increase of 15.2% (US$3,078 or GH ¢17,788) between patients with severe symptoms and those that were critically ill. Deterioration from mild to moderate required US$4,245 (GH ¢24,536) worth of additional resource, representing a 74.4% increase in the cost of treatment.

## Discussion

Our analysis estimates that the average cost of treating a person infected with SARS-COV-2 in Ghana was about US$11,925 (GH¢68,929), which ranged widely from US$282 (GH¢1629) for mild cases managed in home settings to US$23,382 (GH¢135,149) for critically ill patients managed in resource intensive and specialised hospital settings. Thus, the cost of treatment of COVID-19 could increase by at least 20-folds once a patient’s condition warranted to be moved from home management to any type of hospital setting or institutionalised treatment centre.

There are limited number of publicly available works estimating the cost of COVID-19 response, including that of case management which is the thrust of this paper. In one multi-country study underpinned by extrapolation of data from South Africa, Ethiopia and Pakistan, the cost of managing COVID-19 cases was estimated to range from US$147 per patient in home management to as high as US$1082 per case per day for critically ill [20]. For home management, our estimate was, however, 92% higher than that of South Africa (for home-based management of non-symptomatic or mildly ill patients) as reported in the work of Rueda et al. The difference between the two studies was even much higher for severe cases where our estimate was ten times higher. However, estimates of the two studies converged with a difference of less than 3% for the cost of treating critically ill patients (US$1113 per day estimated in the current study versus US$1082 per day in the previous study). Whereas we adopted a bottom-up, point of care resource use data collection approach, Rueda and colleagues were unable to collect primary cost data directly from COVID-19 service delivery points, hence approximated resource use and unit costs from previous works around tuberculosis (TB) or general health services. Thus, the difference in methodological approaches may have contributed to the differences in cost, in addition to the contextual and treatment protocol differences that may exist between Ghana (where the present study was based) and South Africa, Ethiopia and Pakistan (whose contexts were the basis of the previous work).

In a Kenyan study, Barasa et al. estimated the cost of treating COVID-19 cases in Kenya as ranging from US$278 per asymptomatic or mildly ill patient in home management to US$5879 per critically ill patient managed in resource-intense settings [19]; drawing close similarities with current estimates from Ghana’s context for home-based management (US$282 for Ghana versus US$278 for Kenya). However, the estimated cost of treating critically COVID-19 patients in Ghana was more than four times higher than Kenya - a difference that could be attributed to, among other things, an average of twelve days length of stay assumed in the Kenyan study as compared to twenty-one days in the case of Ghana.

Our study showed that once a patient moved from home management to the treatment centre, the average cost of treatment increased by about 20-folds (US$282 to US$5707). A similar costing study found that, in the context of Kenya, home-based care for COVID-19 cases was nine times cheaper than institutional base care scenarios with overheads, staff costs and PPEs being the drivers of the costs difference.

Meanwhile, the present study found that once a patient’s condition deteriorated from mild to moderate, the cost escalated by 155% but the cost-mix shifted from 71% overheads in the case of patients with mild symptoms to 50% for cases with moderate symptoms, while the cost of in-patient care increased from 22 to 35%. Similarly, deteriorating from moderate to severe resulted in more than doubling the costs of treatment but only a marginal difference of 15.2% was found between the cost of patients with severe symptoms versus those that were critically ill.

The current study also estimates that in institutionally managed patients, overhead cost accounted for 63 to 71% of the overall cost of treatment of which 81% were attributable to PPEs, 28% for accommodation and utilities, and 3% for transportation. The cost of drugs accounted for just up to 1% in institutionally managed patients and 5% for patients managed at home. These findings, however, contrasted sharply with those of the Chinese study in which the cost of drugs was observed to be the major cost driver, accounting for 45.1%, of the overall mean [17].

From a cost containment perspective, these findings underline the need for early detection and effective treatment of COVID-19 cases, preferably at home, before any chance of deterioration to the next worst form of the disease state. This is supported by our finding that the cost of treatment could increase by at least 20 folds once a patient moved from home management to any type of institutionalised treatment centre (or hospital), most of which was related to overhead costs. For example, (as shown in Table 2), overhead cost accounted for 63–71% of the cost of treating in hospitals and treatment centres as compared to 6% for those managed at home. The few studies that have reported on the cost of treating COVID-19 also collaborate these findings in the context of Kenya, and broadly low-and middle-income countries. Nevertheless, the substantial cost jumps also raise concerns if there was still room for efficiency gains in the resource use in the management of severe and critically ill patients that may accrue as better evidence on the management of COVID-19 evolved.

Protocol-based cost of illness analysis has potential utility in resource planning and mobilisation in responding to the pandemic and also contributing to cost effectiveness evaluation of the treatment protocol. The results of this analysis were made available to government and other partners through the national COVID-19 response team to be used as part of context specific evidence as in the planning and resource mobilisation in the context of COVID-19 response. However, at the time of preparing the manuscript it was still premature to estimate to estimate the overall impact or contribution of this work to the national COVID-19 response in Ghana. Nevertheless, as this work represents the first attempt at estimating the cost of managing COVID-19 infections in Ghana, its potential utility cannot be overstated.

## Conclusion

From a health system perspective of cost, the clinical management of a COVID-19 patient in Ghana is averagely US$11,925 (GH¢68,929) but ranges widely from US$282 (GH¢1629) for mild/asymptomatic cases who are managed at home to as much as US$23,382 (GH¢135,149) for critically ill patients managed in resource intensive hospital settings. It demonstrates that the cost of treatment is at least 20 times higher in hospital settings or any type of institutionalised treatment centres as compared to home-based management. Therefore, cost savings could be made by early detection and effective treatment of COVID-19 cases, preferably at home, before any chance of deterioration to the next worst form of the disease state, thereby freeing up more resources for other aspects of the fight against the pandemic. Policy makers in Ghana should thus make it a top priority to intensify the early detection and case management of COVID-19 infections.

### Limitations

This study has some inherent limitations that must be considered when using the same for policy or decision making. First, it is worth noting that the estimates reported in this paper are based on Ghana’s COVID-19 Case Management Protocol up to September 2020, after which there might have been some changes, which would likely impact the results if the estimates were to be updated in line with the new protocols. The type of treatment required for COVID-19 as defined in the national treatment protocol at the time of data collection provided no alternative pathways that could be used for sensitivity analysis. Moreover, the cost data was taken from either procurement invoices at the Ministry of Health or the national health insurance reimbursement price list, which also provided no plausible ranges for sensitivity analysis. Under the circumstances, sensitivity analysis was not undertaken in this study which should be considered a limitation.

Second, the costs of treating COVID-19 cases could vary drastically from the time the data was collected in June 2020 given the emergence of new variants and deployment of vaccinations, both of which were not available in Ghana at the time of the analysis. Although, there has not been a major shift in Ghana’s approach to COVID-19 case management, the gaps identified must be considered as a limitation of this work, hence future update of this analysis with real-life data that accounts for the impact of variants and vaccinations would be imperative. Furthermore, the use of drugs, technology, and better risk stratification of patients would likely impact future resource consumption by COVID-19 patients. Based on this, there is a need to continually update the estimation of the costs of COVID-19 case management as the evidence and treatment protocols evolve.

Finally, although every effort was made to use the prevailing market prices as unit costs for resources needed for COVID-19 treatment, volatile pricing resulting from the COVID-19 itself is one factor that could make these estimates quickly outdated. Also, hydroxychloroquine which was part of the treatment protocol was not available on the local market; hence its price was taken from international sources.

Nevertheless, this study, to the best of our knowledge, represents one of the first attempts to undertake bottom-up, point of care resource use data collection approach to estimating the costs of managing COVID-19 cases in Ghana and Africa. The results of this study, although imprecise, provide a reasonable basis for estimating the overall cost of the response and for planning resource needs for fighting the ongoing COVID-19 pandemic in Ghana and other similar contexts.

## Supplementary Information

**Additional file 1.** Table S1. Resource Use and Unit Costs for Critically ill cases of COVID-19. Table S2. Resource Use and Unit Costs for Severe Cases of COVID-19. Table S3. Resource Use and Unit Costs for Moderate Cases of COVID-19. Table S4. Resource Use and Unit Costs for Mild/Asymptomatic Cases of COVID-19 (at hospitals or isolation centers). Table S5. Resource Use and Unit Costs for home-based management of mild/asymptomatic cases.

## Data Availability

The datasets supporting our conclusions are publicly available and will be provided upon request to the corresponding author.

## Abbreviation

WHO: World Health Organization
