# Supplementary material for: The cost of clinical management of SARS-COV-2 (COVID-19) infection by level of disease severity in Ghana: a protocol-based cost of illness analysis
Source: BMC Health Serv Res. 2021 Oct 18;21:1115. doi: 10.1186/s12913-021-07101-z (PMC8521497; doi:10.1186/s12913-021-07101-z)
Supplement: Supplementary file 1 — Additional file 1. Table S1. Resource Use and Unit Costs for Critically ill cases of COVID-19. Table S2. Resource Use and Unit Costs for Severe Cases of COVID-19. Table S3. Resource Use and Unit Costs for Moderate Cases of COVID-19. Table S4. Resource Use and Unit Costs for Mild/Asymptomatic Cases of COVID-19 (at hospitals or isolation centers). Table S5. Resource Use and Unit Costs for home-based management of mild/asymptomatic cases. [file 12913_2021_7101_MOESM1_ESM.docx]

**Supplementary Tables S1 – S5: Resource Use and Unit Costs for various levels of severity of COVID-19**

**Table S1: Resource Use and Unit Costs for Critically ill cases of COVID-19**

| **Resources** | **Quantity per day** | **Average Length of Stay in Hospital-isolation centre/duration of resource Use** | **Unit cost (GH¢)** | **Sub-Total (GH¢)** | **Source of Data** |
| --- | --- | --- | --- | --- | --- |
| Overheads |  |  |  |  |  |
| Patient transportation | 2 times | 1 | 734.00 | 734.00 | GHS transport cost in + state transport fare out |
| Patient accommodation, feeding and utilities per in-patient day | Meals - 3 times per day | 21 | 383.12 | 8,045.52 | National Health Insurance Scheme (NHIS, 2016) - cerebral malaria as proxy |
| Personal Protective Equipment (PPEs) - Doctors | Doctor: 4 PPE per day per patient | 21 | 400.00 | 33,600.00 | Procurement invoices |
| Personal Protective Equipment (PPEs) - Nurses | Nurse: 6 PPE per day per patient | 21 | 400.00 | 50,400.00 | Procurement invoices |
|  |  |  |  |  |  |
| Investigations |  |  |  |  |  |
| COVI-19 test | 3 times per stay | 1 | 367.25 | 1,101.75 | Nugochi Memorial Institute for Medical Research |
| X-rays | Once per stay | 1 | 60.00 | 60.00 | University of Ghana Medical Centre (UGMC) |
| Full Blood Count | 3 times per stay | 1 | 12.04 | 36.12 | NHIS (2016) |
| Computer Tomographic (CT) Scan | Two per stay | 1 | 350.00 | 700.00 | UGMC |
| Blood gases | 3 times per stay | 21 | 11.53 | 726.39 | NHIS (2016) |
| Ultrasound scan (if pregnant) | Once per stay | 1 | 28.29 | 28.29 | NHIS (2016) |
| Electrocardiogram (ECG) | Once per stay (part of continuous monitoring) | 1 | 7.74 | - | NHIS (2016) |
| Chemistry (Liver Function Test, Renal Function Test, Electrolytes) | up to 8 times per stay | 8 | 10.34 | 165.44 | NHIS (2016) |
| Coagulation profile (Bleeding time, platelets, prothrombin time etc) | Once per stay | 1 | 7.70 | 7.70 | NHIS (2016) |
| Medications |  |  |  |  |  |
| Vitamin C | 1 gram per day | 14 | 0.10 | 1.40 | NHIS (2020) |
| Zinc | 20 mg per day | 10 | 0.10 | 1.00 | NHIS (2020) |
| Hydroxychloroquine | 200 mg 3X daily | 10 | 2.14 | 64.20 |  |
| Azithromycin | 500 mg daily | 5 | 3.00 | 15.00 | NHIS (2020) |
| Antibiotics - Ceftriaxone | 2g daily | 5 | 127.16 | 635.80 |  |
| Thrombolytics - Enoxaparin (Rx) | 80mg daily | 14 | 87.10 | 1,219.40 | NHIS (2020) |
| In-patient care |  |  |  |  |  |
| Oxygen | 3,600 litres daily | 14 | 10.00 | 3,360.00 | Greater Accra Regional Hospital |
| Mechanical ventilation | GH¢ 450 per day; monitoring – Gh¢250; central line GH¢ 350 (every 14 days) | 21 | 700.00 | 15,050.00 | Greater Accra Regional Hospital |
| Logistics: Syringes, needles, etc | 30 pieces per stay | 1 | 55.00 | 55.00 | Central Medical Stores (CMS) |
| Staff time |  |  |  |  |  |
| General medical practitioner | Two hours per day | 21 | 54.62 | 2,294.01 | Fair Wages and Salaries Commission (FWSC), 2020 |
| Medical Specialist | Four hours per day | 21 | 71.53 | 6,008.42 | FWSC, 2020 |
| Professional Nurses (General) | Six hours per day | 21 | 37.51 | 4,725.95 | FWSC, 2020 |
| Professional Nurses (Specialised) | Six hours per day | 21 | 37.51 | 4,725.95 | FWSC, 2020 |
| Medical laboratory scientist/technician | 3 hours per stay | 1 | 36.44 | 109.32 | FWSC, 2020 |
| Radiographer/Diagnostic Technician | 30 mins | 1 | 36.44 | 18.22 | FWSC, 2020 |
| Clinical psychologists | 1 hour per stay | 1 | 36.44 | 36.44 | FWSC, 2020 |
| Physiotherapists | 1 hour per stay | 1 | 36.44 | 36.44 | FWSC, 2020 |
| Nutritionist/Dietician | 1 hour per patient per stay | 1 | 36.44 | 36.44 | FWSC, 2020 |
| Emergecy Medical Technician | 16 hours per stay | 1 | 16.08 | 257.28 | FWSC, 2020 |
| Orderlies (cleaning of patient and staff areas) | Three hours per day per patient | 21 | 9.85 | 620.54 | FWSC, 2020 |
| Pharmacist | Two hours per patient per entire stay | 1 | 36.44 | 72.88 | FWSC, 2020 |
| Kitchen Staff | Two hours per patient per entire stay | 1 | 9.36 | 18.72 | FWSC, 2020 |
| Administrative /Health Information Staff -Documentation | Two hours per patient per entire stay | 1 | 23.87 | 47.74 | FWSC, 2020 |
| Security | Throughout | 21 | 12.74 | 133.74 | FWSC, 2020 |
|  |  |  |  | **135,149.10** |  |

**Table S2: Resource Use and Unit Costs for Severe Cases of COVID-19**

| **Resources** | **Quantity per day** | **Average Length of Stay in Hospital-isolation centre/duration of resource Use** | **Unit cost (GH¢)** | **Sub-Total (GH¢)** | **Source of Data** |
| --- | --- | --- | --- | --- | --- |
| Overheads |  |  |  |  |  |
| Patient transportation | 2 times | 1 | 734.00 | 734.00 | GHS transport cost in + state transport fare out |
| Patient accommodation, feeding and utilities per in-patient day | Meals - 3 times per day | 19 | 383.12 | 7,279.28 | NHIS (2016) |
| Personal Protective Equipment (PPEs) - Doctors | Doctor: 4 PPEs per day per patient | 19 | 400.00 | 30,400.00 | Procurement invoices |
| Personal Protective Equipment (PPEs) - Nurses | Nurse: 6 PPEs per day per patient | 19 | 400.00 | 45,600.00 | Procurement invoices |
| Investigations |  |  |  |  |  |
| COVI-19 test | 3 times per stay | 1 | 367.25 | 1,101.75 | Nugochi Memorial Institute for Medical Research |
| X-rays | Once per stay | 1 | 60.00 | 60.00 | NHIS (2016) |
| Full Blood Count | Twice per stay | 1 | 12.04 | 24.08 | NHIS (2016) |
| Computer Tomographic (CT) Scan | Once per stay | 1 | 350.00 | 350.00 | NHIS (2016) |
| Blood gases |  |  | 11.53 | - | NHIS (2016) |
| Ultrasound scan (if pregnant) | Once per stay | 1 | 28.29 | 28.29 | NHIS (2016) |
| Electrocardiogram (ECG) | Once per stay | 1 | 7.74 | 7.74 | NHIS (2016) |
| Chemistry (Liver Function Test, Renal Function Test, Electrolytes) | Twice per stay | 1 | 10.34 | 20.68 | NHIS (2016) |
| Coagulation profile | Once per stay | 1 | 7.70 | 7.70 | NHIS (2016) |
| Medications |  |  |  |  |  |
| Vitamin C | 1 gram per day | 14 | 0.10 | 1.40 | NHIS (2020) |
| Zinc | 20 mg per day | 10 | 0.10 | 1.00 | NHIS (2020) |
| Hydroxychloroquine | 200 mg 3X daily | 10 | 2.14 | 64.20 | Open Source |
| Azythromycin | 500 mg daily | 5 | 3.00 | 15.00 | NHIS (2020) |
| Antibiotics - Ceftriaxone | 2g daily | 5 | 127.16 | 635.80 | NHIS (2020) |
| Thrombolytics - Enoxaparin (Rx) | 80mg daily | 5 | 87.10 | 435.50 | NHIS (2020) |
| In-patient care |  |  |  |  |  |
| Oxygen | 3,600 liters daily | 14 | 10.00 | 3,360.00 | Greater Accra Regional Hospital |
| Mechanical ventilation | Based on need | 14 | 700.00 | 9,800.00 | Greater Accra Regional Hospital |
| Logistics: Syringes, needles, etc | 30 pieces per stay | 1 | 55.00 | 55.00 | Central Medical Stores (CMS) |
| Staff time |  |  |  |  |  |
| General medical practitioner | Two hours per day | 19 | 54.62 | 2,075.53 | FWSC, 2020 |
| Medical Specialist (ISCO xxxx) | Four hours per day | 19 | 71.53 | 5,436.19 | FWSC, 2020 |
| Professional Nurses (General) | Six hours per day | 19 | 37.51 | 4,275.86 | FWSC, 2020 |
| Professional Nurses (Specialised) | Six hours per day | 19 | 37.51 | 4,275.86 | FWSC, 2020 |
| Nursing Associate professionals/Auxiliary Nurses (ISCO 2222) |  |  | 17.19 | - | FWSC, 2020 |
| Medical laboratory scientist/technician | 3 hours per stay | 1 | 36.44 | 109.32 | FWSC, 2020 |
| Radiographer/Diagnostic Technician | 30 mins | 1 | 36.44 | 18.22 | FWSC, 2020 |
| Clinical psychologists | 1 hour per stay | 1 | 36.44 | 36.44 | FWSC, 2020 |
| Physiotherapists | 1 hour per stay | 1 | 36.44 | 36.44 | FWSC, 2020 |
| Nutritionist/Dietician | 1 hour per patient per stay | 1 | 36.44 | 36.44 | FWSC, 2020 |
| Emergecy Medical Technician | 16 hours per stay | 1 | 16.08 | 257.28 | FWSC, 2020 |
| Orderlies (cleaning of patient and staff areas) | Three hours per day per patient | 19 | 9.85 | 561.44 | FWSC, 2020 |
| Pharmacist | Two hours per patient per entire stay | 1 | 36.44 | 72.88 | FWSC, 2020 |
| Kitchen Staff | Two hours per patient per entire stay | 1 | 9.36 | 18.72 | FWSC, 2020 |
| Administrative /Health Information Staff -Documentation | Two hours per patient per entire stay | 1 | 23.87 | 47.74 | FWSC, 2020 |
| Security | Throughout | 19 | 12.74 | 121.00 | FWSC, 2020 |
|  |  |  |  | **117,360.79** |  |

**Table S3: Resource Use and Unit Costs for Moderate Cases of COVID-19**

| **Resources** | **Quantity per day** | **Average Length of Stay in Hospital-isolation centre/duration of resource Use** | **Unit cost (GH¢)** | **Sub-Total (GH¢)** | **Source of Data** |
| --- | --- | --- | --- | --- | --- |
| Overheads |  |  |  |  |  |
| Patient transportation | 2 times | 1 | 734.00 | 734.00 | GHS transport cost in + state transport fare out |
| Patient accommodation, feeding and utilities per in-patient day | Meals - 3 times per day | 19 | 383.12 | 7,279.28 | NHIS (2016) |
| Personal Protective Equipment (PPEs) - Doctors | Doctor: 2 PPE per day per patient | 19 | 400.00 | 15,200.00 | Procurement invoices |
| Personal Protective Equipment (PPEs) - Nurses | Nurse: 3 PPE per day per patient | 19 | 400.00 | 22,800.00 | Procurement invoices |
| Investigations |  |  |  |  |  |
| COVI-19 test | 2 times per stay | 2 | 367.25 | 1,469.00 | Nugochi Memorial Institute for Medical Research |
| X-rays | Once per stay | 1 | 60.00 | 60.00 | University of Ghana Medical Centre (UGMC) |
| Full Blood Count | Twice per stay | 2 | 12.04 | 48.16 | NHIS (2016) |
| Computer Tomographic (CT) Scan | Once per stay | 1 | 350.00 | 350.00 | NHIS (2016) |
| Ultrasound scan (if pregnant) | Once per stay | 1 | 28.29 | 28.29 | NHIS (2016) |
| Electrocardiogram (ECG) | Once per stay | 1 | 7.74 | 7.74 | NHIS (2016) |
| Medications |  |  |  |  |  |
| Vitamin C | 1 gram per day | 14 | 0.10 | 1.40 | NHIS (2020) |
| Zinc | 20 mg per day | 10 | 0.10 | 1.00 | NHIS (2020) |
| Hydroxychloroquine | 200 mg 3X daily | 10 | 2.14 | 64.20 | Open Source |
| Azythromycin | 500 mg daily | 5 | 3.00 | 15.00 | NHIS (2020) |
| Thrombolytics | 40mg daily | 5 | 87.10 | 435.50 | NHIS (2020) |
| In-patient care |  |  |  |  |  |
| Logistics: Syringes, needles, etc | 30 pieces per stay | 1 | 55.00 | 55.00 | Central Medical Stores (CMS) |
| Staff time |  |  |  |  |  |
| General medical practitioner | Two hours per day | 19 | 54.62 | 2,075.53 | FWSC, 2020 |
| Medical Specialist | One hour per day | 19 | 71.53 | 1,359.05 | FWSC, 2020 |
| Professional Nurses (General) | Three hours per day | 19 | 37.51 | 2,137.93 | FWSC, 2020 |
| Professional Nurses (Specialised) | One hour per day | 19 | 37.51 | 712.64 | FWSC, 2020 |
| Nursing Associate professionals/Auxiliary Nurses | Three hours per day | 19 | 17.19 | 979.97 | FWSC, 2020 |
| Medical laboratory scientist/technician | 3 hours per stay | 1 | 36.44 | 109.32 | FWSC, 2020 |
| Radiographer/Diagnostic Technician | 30 mins | 1 | 36.44 | 18.22 | FWSC, 2020 |
| Clinical psychologists | 1 hour per stay | 1 | 36.44 | 36.44 | FWSC, 2020 |
| Physiotherapists | 30 mins per stay | 1 | 36.44 | 18.22 | FWSC, 2020 |
| Nutritionist/Dietician | 1 hour per patient @ per stay | 1 | 36.44 | 36.44 | FWSC, 2020 |
| Emergecy Medical Technician | 16 hours per stay | 1 | 16.08 | 257.28 | FWSC, 2020 |
| Orderlies (cleaning of patient and staff areas) | Three hours per day per patient | 19 | 9.85 | 561.44 | FWSC, 2020 |
| Pharmacist | Two hours per patient per entire stay | 2 | 36.44 | 145.76 | FWSC, 2020 |
| Kitchen Staff | Two hours per patient per entire stay | 19 | 9.36 | 355.70 | FWSC, 2020 |
| Administrative /Health Information Staff -Documentation | Two hours per patient per entire stay | 1 | 23.87 | 47.74 | FWSC, 2020 |
| Security | Throughout | 19 | 12.74 | 121.00 | FWSC, 2020 |
|  |  |  |  | **57,521.25** |  |

**Table S4: Resource Use and Unit Costs for Mild/Asymptomatic Cases of COVID-19 (at hospitals or isolation centers)**

| **Resources** | **Quantity per day** | **Quantity per day (calc)** | **Average Length of Stay in Hospital-isolation centre/duration of resource Use** | **Unit cost (GH¢)** | **Sub-Total (GH¢)** | **Source of Data** |
| --- | --- | --- | --- | --- | --- | --- |
| Overheads |  |  |  |  |  |  |
| Patient transportation | 2 times | 1 | 1 | 734.00 | 734.00 | GHS transport cost in + state transport fare out |
| Patient accommodation, feeding and utilities per in-patient day | Meals @3 times per day | 1 | 19 | 383.12 | 7,279.28 | NHIS (2016) |
| Personal Protective Equipment (PPEs) - Doctors | Doctor: 1 PPE per day per patient | 1 | 19 | 400.00 | 7,600.00 | Procurement invoices |
| Personal Protective Equipment (PPEs) - Nurses | Nurse: 2 PPE per day per patient | 2 | 19 | 400.00 | 15,200.00 | Procurement invoices |
| Investigations |  |  |  |  |  |  |
| COVI-19 test | 2 times per stay | 2 | 1 | 367.25 | 734.50 | Nugochi Memorial Institute for Medical Research |
| X-rays | Once per stay | 1 | 1 | 60.00 | 60.00 | University of Ghana Medical Centre (UGMC) |
| Full Blood Count | Twice per stay | 2 | 1 | 12.04 | 24.08 | NHIS (2016) |
| Ultrasound scan (if pregnant) | Once per stay | 1 | 1 | 28.29 | 28.29 | NHIS (2016) |
| Medications |  |  |  |  |  |  |
| Vitamin C | 1 gram per day | 1 | 14 | 0.10 | 1.40 | NHIS (2020) |
| Zinc | 20 mg per day | 1 | 10 | 0.10 | 1.00 | NHIS (2020) |
| Hydroxychloroquine | 300 mg 3X daily | 3 | 10 | 2.14 | 64.20 | Open Source |
| Azythromycin | 500 mg daily | 1 | 5 | 3.00 | 15.00 | NHIS (2020) |
| In-patient care |  |  |  |  |  |  |
| Staff time |  |  |  |  |  |  |
| General medical practitioner | One hour per day | 1 | 1 | 54.62 | 54.62 | FWSC, 2020 |
| Professional Nurses (General) | Two hours per day | 2 | 2 | 37.51 | 150.03 | FWSC, 2020 |
| Professional Nurses (Specialised) | One hour per day | 1 | 1 | 37.51 | 37.51 | FWSC, 2020 |
| Medical laboratory scientist/technician | 2 hours per stay | 2 | 1 | 36.44 | 72.88 | FWSC, 2020 |
| Radiographer/Diagnostic Technician | 30 mins | 0.5 | 1 | 36.44 | 18.22 | FWSC, 2020 |
| Clinical psychologists | I hour per stay | 1 | 1 | 36.44 | 36.44 | FWSC, 2020 |
| Physiotherapists |  | 0 | 0 | 36.44 | - | FWSC, 2020 |
| Nutritionist/Dietician | 30 mins per patient @ per stay | 0.5 | 1 | 36.44 | 18.22 | FWSC, 2020 |
| Emergency Medical Technician | 16 hours per stay | 16 | 1 | 16.08 | 257.28 | FWSC, 2020 |
| Orderlies (cleaning of patient and staff areas) | Two hours per day per patient | 2 | 19 | 9.85 | 374.29 | FWSC, 2020 |
| Pharmacist | One hour per patient per entire stay | 1 | 1 | 36.44 | 36.44 | FWSC, 2020 |
| Kitchen Staff | Two hours per patient per entire stay | 2 | 1 | 9.36 | 18.72 | FWSC, 2020 |
| Administrative /Health Information Staff -Documentation | Two hours per patient per entire stay | 2 | 1 | 23.87 | 47.74 | FWSC, 2020 |
| Security | Throughout | 0.5 | 19 | 12.74 | 121.00 | FWSC, 2020 |
|  |  |  |  |  | **32,985.14** |  |

**Table S5: Resource Use and Unit Costs for home-based management of mild/asymptomatic cases**

| **Resources** | **Quantity per day** | **Average Length of Stay in Hospital-isolation centre/duration of resource Use** | **Unit cost (GH¢)** | **Sub-Total (GH¢)** | **Source of Data** |
| --- | --- | --- | --- | --- | --- |
| Overheads |  |  | - | - |  |
| Thermometre | 1 | 1 | 100.00 | 100.00 | Procurement invoices |
| Investigations |  |  | - | - |  |
| COVI-19 test | 2 times per stay | 1 | 367.25 | 734.50 | Nugochi Memorial Institute for Medical Research |
| Ultrasound scan (if pregnant) | Once per stay | 1 | 28.29 | 28.29 | University of Ghana Medical Centre (UGMC) |
| Medications |  |  | - | - |  |
| Vitamin C | 1 gram per day | 14 | 0.10 | 1.40 | NHIS (2020) |
| Zinc | 20 mg per day | 10 | 0.10 | 1.00 | NHIS (2020) |
| Hydroxychloroquine | 300 mg 3X daily | 10 | 2.14 | 64.20 | Open Source |
| Azythromycin | 500 mg daily | 5 | 3.00 | 15.00 | NHIS (2020) |
| In-patient care |  |  |  |  |  |
| Staff time |  |  |  |  |  |
| General medical practitioner | 16 Minutes per day | 19 | 54.62 | 280.20 | FWSC, 2020 |
| Professional Nurses (General) | 16 Minutes per day | 19 | 37.51 | 192.41 | FWSC, 2020 |
| Medical laboratory scientist/technician | 2 hours per stay | 1 | 36.44 | 72.88 | FWSC, 2020 |
| Clinical psychologists | 1 hour per stay | 1 | 36.44 | 36.44 | FWSC, 2020 |
| Nutritionist/Dietician | 30 minutes per patient per stay | 1 | 36.44 | 18.22 | FWSC, 2020 |
| Pharmacist | 1 hour per patient per entire stay | 1 | 36.44 | 36.44 | FWSC, 2020 |
| Administrative /Health Information Staff -Documentation | 2 hours per patient per entire stay | 1 | 23.87 | 47.74 | FWSC, 2020 |
|  |  |  |  | **1,628.72** |  |
